# Visual function loss in fungal sphenoid sinusitis: clinical characteristics and outcomes

**DOI:** 10.1038/s41598-024-59107-2

**Published:** 2024-04-15

**Authors:** Fei Chen, Yonghui Shao, Qian Huang, Yue Chen, Bentao Yang, Libin Jiang

**Affiliations:** 1 Beijing Tongren Eye Center, Beijing Tongren Hospital, Capital Medical University, 1 Dongjiaomin Lane, Dongcheng District, Beijing, 100010 China; 2 Department of Ophthalmology, Tengzhou Central People’s Hospital, No.181 Xingtan Road, Tengzhou City, 277500 Shandong Province China; 3 Department of Otolaryngology Head and Neck Surgery, Beijing Tongren Hospital, Capital Medical University, Beijing, 100010 China; 4 Department of Radiology, Beijing Tongren Hospital, Capital Medical University, Beijing, 100010 China

**Keywords:** Fungal sphenoid sinusitis, Aspergillus, Mucormycosis, Visual damage, Clinical features, Visual outcomes, Fungal infection, Optic nerve diseases

## Abstract

Potentially fatal fungal sphenoid sinusitis (FSS) causes visual damage. However, few studies have reported on its visual impairment and prognosis. Five hundred and eleven FSS patients with ocular complications treated at Beijing Tongren Hospital were recruited and clinical features and visual outcomes were determined. Thirty-two of the 511 patients (6%) had visual impairment, with 13 and 19 patients having invasive and noninvasive FSS, respectively. Eighteen patients (56.25%) had diabetes and 2 patient (6.25%) had long-term systemic use of antibiotics (n = 1) and corticosteroids (n = 1). All patients had visual impairment, which was more severe in invasive FSS than in noninvasive FSS. Bony wall defects and sclerosis were observed in 19 patients (59.38%), and 11 patients (34.38%) had microcalcification in their sphenoid sinusitis on computed tomography (CT). After a 5-year follow-up, three patients (9.38%) died. Patients with noninvasive FSS had a higher improvement rate in visual acuity than their counterparts. In the multivariate analysis, sphenoid sinus wall sclerosis on CT was associated with better visual prognosis. FSS can cause vision loss with persistent headaches, particularly in those with diabetes. CT showed the sphenoid sinus wall sclerosis, indicating a better visual prognosis in FSS with visual impairment.

## Introduction

Fungi occur widely in nature as fungal spores, which can be inhaled into the nasal cavity, leading to colonization of the upper respiratory tract mucosa^1^. They can be engulfed and destroyed by tissue macrophages and neutrophils. However, with impaired host immunity, spores can germinate into hyphae, invade host mucosa, and cause invasive disease^2^. Fungal sphenoid sinusitis (FSS) is an uncommon and potentially fatal disease. The causative pathogen or disease-related inflammation affects the adjacent important structures, including the cavernous sinus, optic nerve, internal carotid artery, and cranial nerves III, IV, V, and VI, causing severe orbital or intracranial complications^3^. The early symptoms of FSS are atypical, which can manifest as head and face pain and swelling, often resembling bacterial infections and idiopathic inflammatory conditions of the sinus or orbit. Nasal symptoms are frequently absent^4–6^. In addition to the symptoms of sinusitis, FSS often combined with ocular complications, manifest as monocular or binocular visual impairment^7^. This infection’s rarity and vague symptoms, make its identification challenging, sometimes resulting in misdiagnosis and inappropriate corticosteroid treatment, which may worsen the condition. Furthermore, there are limited reports of FSS patients with visual damage. They mainly focus on the clinical manifestations of the disease and lack an analysis of the treatment results and factors associated with the long-term visual prognosis. This study aimed to present the clinical presentations, imaging features, treatment, and outcomes in FSS with visual impairment and analyze the factors associated with visual prognosis.

## Materials and methods

This retrospective study included patients with ocular complications caused by FSS treated at Beijing Tongren Hospital, Capital Medical University, between January 2006 and December 2020. All study participants provided informed consent. For patients who had died, informed consent was obtained from their legal guardians. Patients with fungal aggregation in the sphenoid sinus, confirmed by histopathology, were divided into invasive and non-invasive groups, according to the presence of fungal components in the mucosa, submucosa, vascular tissue, or bone^8^. The noninvasive subtypes included allergic fungal sinusitis (AFS) and fungal ball. AFS refers to the presence of allergic mucin and sparse fungal hyphae in one sinus cavity. Fungal balls are described as the presence of a noninvasive conglomeration of dense fungal hyphae within the affected sinuses. The invasive subtypes included acute invasive fungal sinusitis (≤ 4 weeks), chronic invasive fungal sinusitis (> 4 weeks), and granulomatous invasive fungal sinusitis^9^. Fungal etiology was determined through tissue histopathology or culture. Data collected included those on medical history, demographics, time interval between ocular symptom occurrence and surgery, clinical presentation, imaging features, treatment, and outcomes. Histopathological findings, culture results, and neutrophil counts were also reviewed.

According to Schulze-Bonsel et al.^10^, visual acuity on the Snellen chart was converted to the log of the minimum angle of resolution (log MAR) scale for quantitative assessment. Count fingers (CF), light perception (LP), and no LP (NLP) were substituted with 1.85, 2.70, and 3.00 log MAR^11^, respectively. Improvement in visual acuity was evaluated qualitatively based on the best-corrected Snellen visual acuity (BCVA) changes measured during a follow-up period of 6 months to 5 years. When the preoperative BCVA was 20/200 or better, treatment success was defined as an improvement over two lines of the visual acuity chart. Preoperative BCVA worse than 20/200 was grouped into five grades: NLP, LP, CF, 20/1000, and 20/400. The improvement in postoperative BCVA by one or more grades compared with preoperative BCVA was considered significant.

All statistical analyses were completed using IBM SPSS V.26.0 (Armonk, NY, USA). Differences in clinical manifestations between patients with invasive and noninvasive FSS were tested using Pearson’s chi-square test, Fisher’s exact test, and the Mann–Whitney U test. Regression analysis of visual prognosis was performed using generalized estimating equation (GEE) models accounting for inter-eye dependencies. Baseline variables with a *P*-value < 0.05 in univariate analysis were included in the multivariate analysis. Statistical significance was defined as *P* < 0.05.

### Ethics declarations

This study was approved by the Ethics committee of Beijing Tongren Hospital, Capital Medical University (Approval No. TRECKY2020-0425) and followed the principle of the declaration of Helsinki.

## Results

Of 511 FSS patients, we identified visual impairment in 32 patients (6%), including 21 women and 11 men. The mean age was 62.81 years (29–83 years) (Table 1).

**Table 1:** Clinical characteristics of ocular complications of fungal sphenoid sinusitis.

Variable	No	%
Gender		
Male	11	34.38
Female	21	65.63
Age (years) Mean ± SD (range)	62.81 ± 12.02 (29–83)	
Pathologic diagnosis		
Invasive fungal sphenoid sinusitis	13	40.63
Noninvasive fungal sphenoid sinusitis	19	59.37
Types of Fungi		
* Aspergillus*	28	87.50
* Mucor*	4	12.50
Underlying disease	20	62.50
Diabetes	18	56.25
Long-term use of corticosteroids/antibiotic	2	6.25
None	12	37.50
Visual loss	32	
Unilateral	26	81.25
Bilateral	6	18.75
Ophthalmic symptoms		
Diplopia	5	15.63
Restricted eye movement	8	25.00
Blepharoptosis	10	31.25
Proptosis	4	12.50
Nasal complaints	10	31.25
Nasal obstruction	5	15.63
Rhinorrhea	7	21.88
Hyposmia	3	9.38
Headache	26	81.25
Mortality		
Invasive fungal sphenoid sinusitis	3	15.80
Noninvasive fungal sphenoid sinusitis	0	0
Neutrophils (Mean ± SD)	4.71 ± 2.25	
Diabetic patients	5.35 ± 2.47	
Non-diabetic patients	3.98 ± 1.79	

There were 18 patients with diabetes and one with breast cancer. One patient was taking antibiotics for chronic bronchiectasis and one was taking long-term corticosteroids for rheumatoid arthritis. Complete blood counts were within normal limits for all patients. By definition, 13 patients showed tissue invasion and one patient showed eosinophils and scanty fungal elements in the biopsy specimen. The fungal etiology was mucormycosis and Aspergillus in four patients (12.5%) and 28 patients (87.5%), respectively. The mean time from symptom onset to diagnosis in noninvasive FSS was longer than that in invasive FSS (*P *= 0.049, Table 2).

**Table 2:** Comparison of clinical features between invasive fungal sphenoid sinusitis and noninvasive fungal sphenoid sinusitis.

	Invasive fungal sphenoid sinusitis (n = 13)	Noninvasive fungal sphenoid sinusitis (n = 19)	*P* value
Age (years)	Mean ± SD 61.38 ± 7.03	Mean ± SD 63.79 ± 14.61	0.540^a^
Gender (M:F)	5:8	6:13	0.051^b^
Time interval (month)	1.00 ± 0.62	2.71 ± 2.86	**0.049**^**c**^
Headache (n, %)	10 (76.9)	16 (84.2)	0.666^b^
DM (n, %)	7 (53.8)	11 (57.9)	1.000^b^
Diplopia (n, %)	2 (15.4)	3 (15.8)	1.000^b^
Motility deficits (n, %)	6 (46.2)	2 (10.5)	**0.038**^b^
Blepharoptosis (n, %)	7 (53.8)	3 (15.8)	**0.049**^b^
Proptosis (n, %)	3 (23.1)	1 (5.3)	0.279^b^
Visual loss (eyes)	14	24	
Unilateral	12	14	
Bilateral	1	5	
Initial BCVA (Log MAR)	Mean ± SD 2.60 ± 0.79	Mean ± SD 1.12 ± 1.17	**< 0.001**^**d**^
Postoperative BCVA (Log MAR)	Mean ± SD 1.97 ± 1.17	Mean ± SD 0.72 ± 0.99	**0.001**^**d**^
Visual acuity improvement rate (n, %)	5 (35.71)	17 (70.83)	**0.026**^**d**^
CT findings			
Intralesional calcification (n, %)	2 (15.4)	9 (47.37)	0.128^b^
Sinus wall erosion (n, %)	10 (76.9)	9 (47.37)	0.147^b^
Sinus wall sclerosis (n, %)	7 (53.8)	12 (63.16)	0.720^b^
Neutrophils	Mean ± SD 5.07 ± 1.94	4.49 ± 2.31	0.832^c^

Significant values are in bold.

BCVA, best-corrected visual acuity; Log MAR, logarithm of the minimum angle of resolution; Time Interval, the mean time from onset of decreased vision to diagnosis; *p*^a^: Independent-Sample T Test; *p*^b^: Fisher exact test. *p*^**c**^: Mann–Whitney U test; *p*^d^: generalized estimating equation (GEE).

### Clinical manifestation

**Figure 1:**
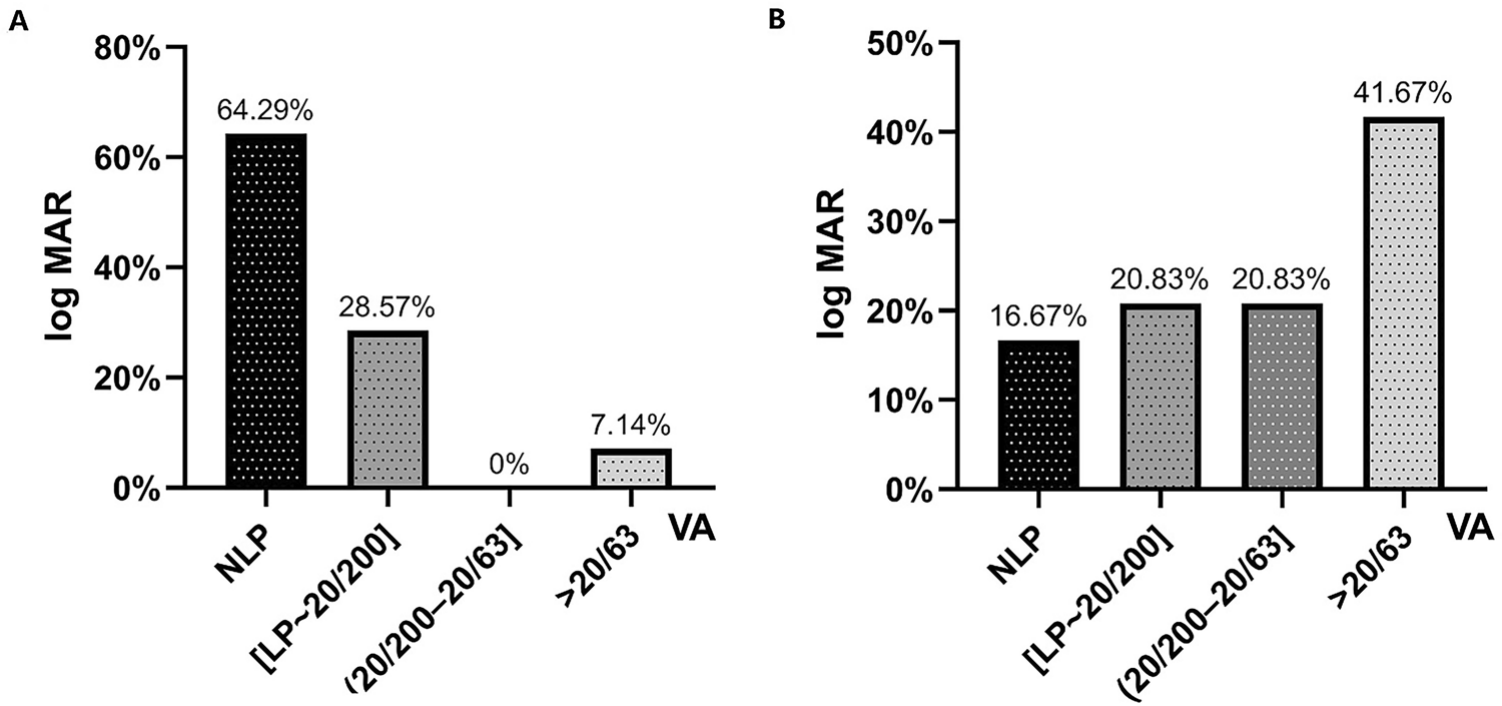
Decreased vision secondary to invasive (**A**) and noninvasive (**B**) fungal sphenoid sinusitis (FSS).

All patients had decreased visual acuity, and it was bilateral in six patients (18.75%). The visual impairment in patients with invasive FSS was more serious than that in patients with noninvasive FSS (*P *< 0.001, Table 2). Nine patients (nine eyes [64.3%]) with invasive FSS ultimately declined to NLP and three patients (four eyes [16.7%]) with noninvasive FSS had NLP (*P* = 0.005, Fig. 1). Other ocular symptoms included diplopia (5, 15.63%), motility deficits (8, 25%), blepharoptosis (10, 31.25%), and proptosis (4, 12.5%). The prevalence of motility deficits and blepharoptosis in the invasive FSS group was significantly higher than that in the noninvasive FSS group (Table 2). Of the 18 patients with diabetes, 14 had diabetic retinopathy (DR): two cases showed mild non-proliferative DR without macular edema, and 12 showed moderate non-proliferative DR with non-center-involved macular edema. The pallor of the optic disc was noted in eight eyes, and mild optic disc swelling was observed in two eyes, and there was no abnormality in the fundus of other patients. Headache was a presenting symptom in 26 patients (81.25%), and only 10 (23%) had nasal complaints (Table 1).

### Imaging features

**Figure 2:**
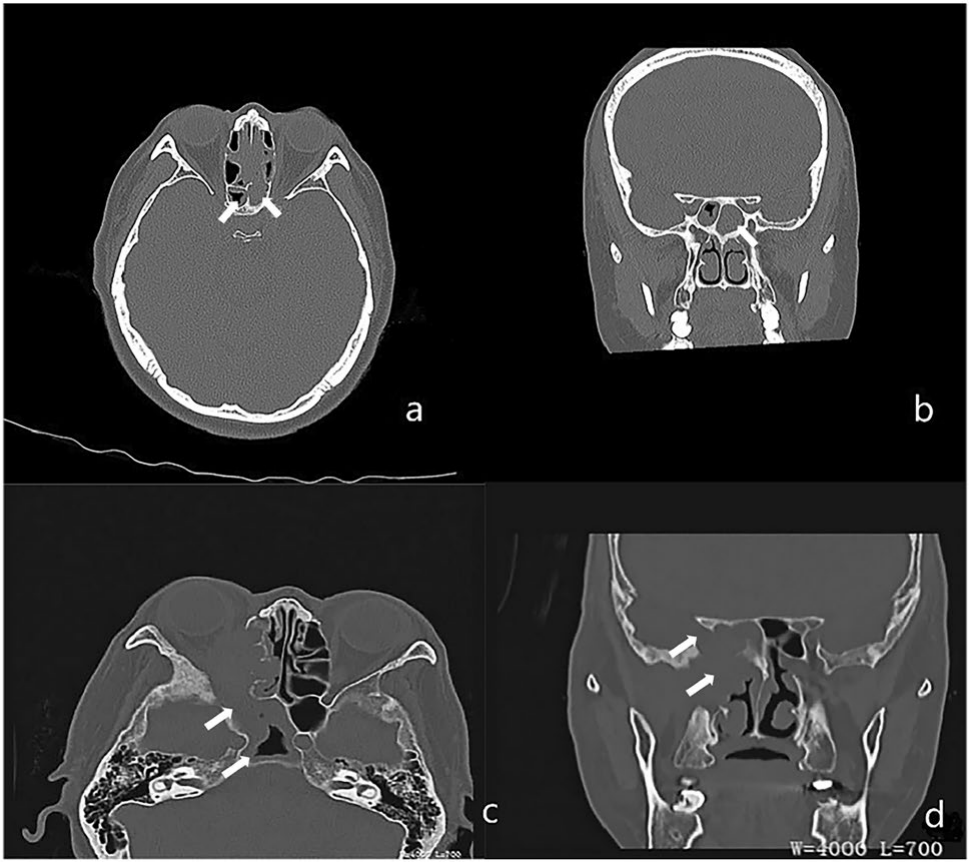
Computed tomography (CT) findings of fungal sphenoid sinusitis (FSS). (**a**, **b**) show complete opacity of the left sphenoid sinus with calcification (white arrow), part opacity of the right sphenoid sinus in noninvasive FSS (white arrow). (**c**, **d**) show complete opacity of the right sphenoid sinus with surrounding bone destruction (white arrow) in invasive FSS.

Computed tomography (CT) of the paranasal sinus or orbit showed partial or complete opacification and mucosal thickening of the affected sinus in these patients. We observed microcalcification in 11 cases (34.38%); its prevalence in the noninvasive FSS group was three times that in the invasive FSS group (n = 9, 47.4% vs. n = 2, 15.4%, Table 2). However, the difference was not significant. Bone changes were noted in 28 patients: 19 (59.38%) showed bony erosion, 19 (59.38%) showed bony thickening and sclerosis. There was no significant difference between the invasive and noninvasive FSS groups (Table 2, Fig. 2).

**Figure 3:**
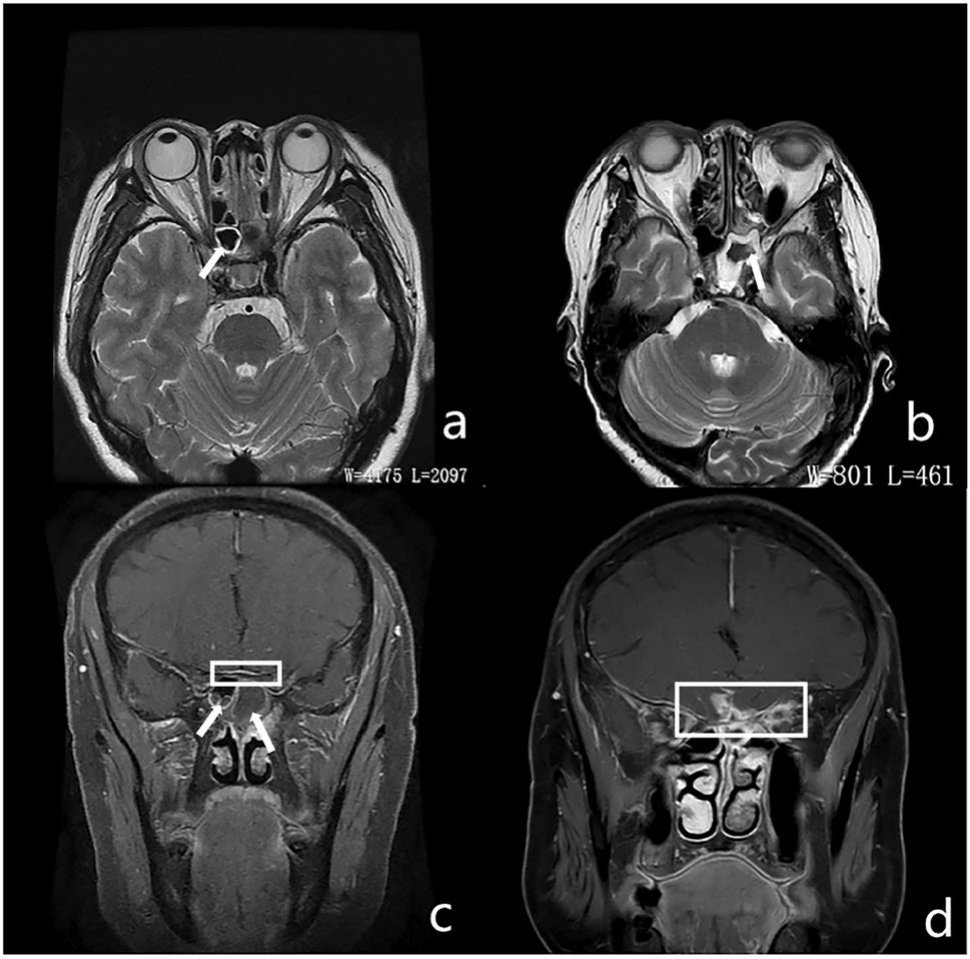
MRI findings of FSS. (**a**, **b**) show low signal of right sphenoid sinus on T2WI (white arrow), peripheral mucosal thickening and linear enhancement (white arrow). Irregular low T2 signal shadows can be seen in the left sphenoid sinus (white arrow). (**c**, **d**) show the hypointensity in bilateral sphenoid sinus on T1WI , without enhancement (white arrow). The lesion invades the skull base, and enhancement can be seen in the adjacent skull base membrane (white box).

**Figure 4:**
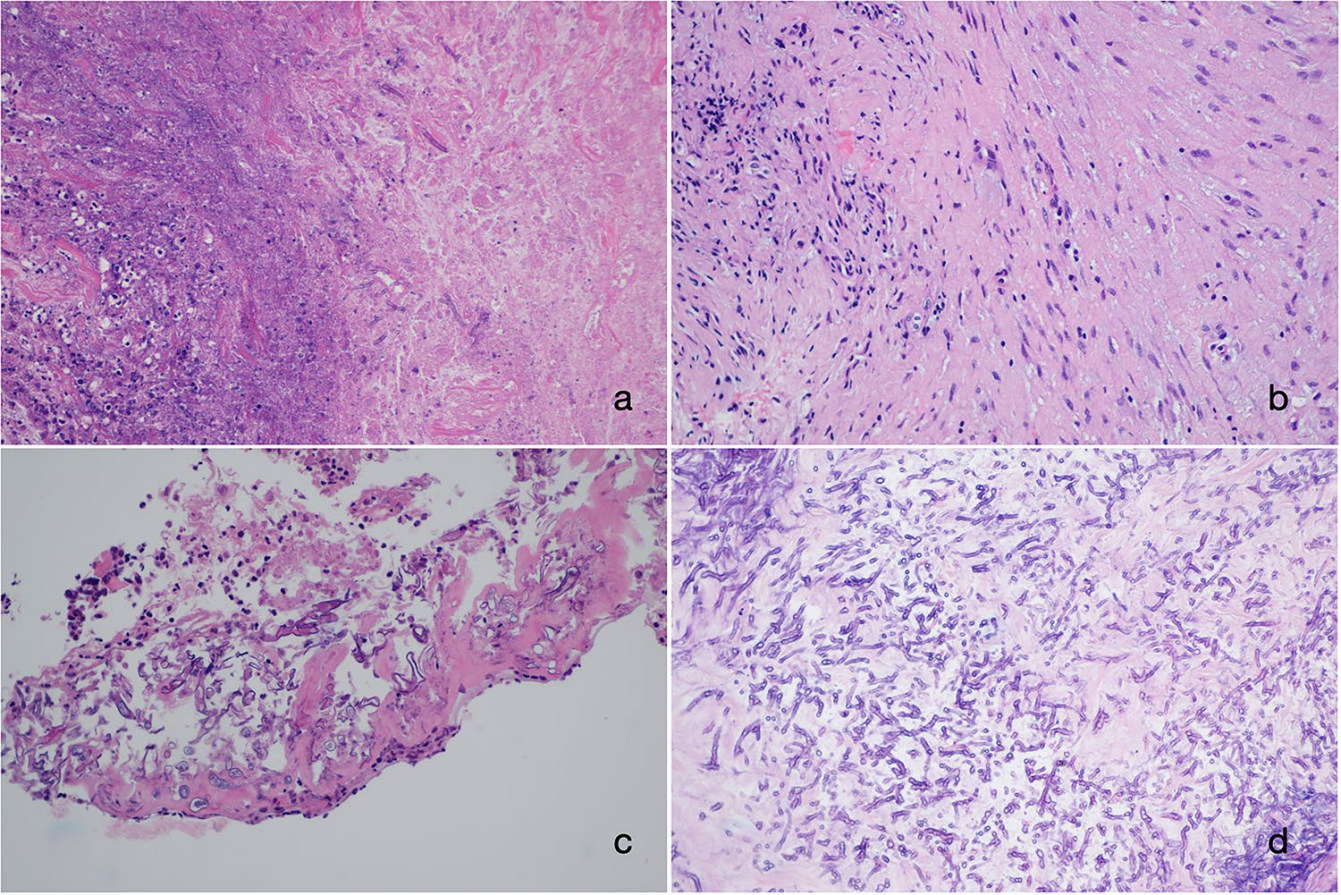
Microphotograph of histopathology specimen (hematoxylin and eosin stain, × 200). (**a**) shows tissue necrosis with varying numbers of inflammatory cells in the early stage, (**b**) shows prominent fibrosis in the late stage. (**c**, **d**) show cytomorphology of Mucorales (**c**) and Aspergillus (**d**).

Magnetic resonance imaging (MRI) of the paranasal sinus or orbit was performed in 17 cases of noninvasive FSS and 12 cases of invasive FSS. On T1-weighted images (T1WI), eight (8/17, 47.06%) of the noninvasive fungal masses were hyperintense (compared with brain parenchyma) and seven (7/17, 41.18%) were hypointense, two (11.76%) of which were isointense. All cases were marked hypointense on T2-weighted images (T2WI), of which, two were uneven and accompanied by an isointense signal. On T1WI, six cases (6/12, 50%) of invasive FSS showed homogeneous hypointensity and six (6/12, 50%) were isointense, of which, one had an accompanied small amount of hypointensity. The lesions showed variable signal intensity on T2WI: three cases (3/12, 25%) were mainly hypointense with different numbers and shapes of hyperintensity (Fig. 3), the duration of symptoms were > 6 months, surgery confirmed that the lesions had prominent fibrous tissue hyperplasia; nine cases (9/12, 75%) showed hyperintensity, the duration of symptoms were < 5 months, histopathology showed tissue necrosis with varying numbers of inflammatory cells, of which five cases had granulomas (Fig. 4). The mucosa around the lesion was significantly thickened with isointensity on T1WI and hyperintensity on T2WI. There were 17 cases with infiltration of the orbital apex, 14 of which involved the cavernous sinus. The optic nerve showed hyperintensity on T2WI in four cases (4/29, 13.79%). Thickening and enhancement of the optic nerve sheath were observed in four cases (4/29, 13.79%).

### Treatment and outcome

Excision of the necrotic tissues and sinuses was performed in all patients, and four patients (12.5%) had received glucocorticoid treatment preoperatively because they were misdiagnosed as having optic neuritis. Postoperatively, 13 patients were treated with antifungal drugs. Two patients were treated with intravenous liposomal amphotericin B, and 11 with intravenous voriconazole combined with irrigation of the involved paranasal sinuses with amphotericin B. Three patients died of progressive disease owing to intracranial spread 1, 3, and 5 months postoperatively, respectively. All of them were found to have a mucormycosis infection. Of the remaining cases, patients with ptosis or limited eye movement improved partially or even completely. The visual acuity of 22 eyes from 19 patients (57.89%) improved significantly, whereas there was no improvement in 16 eyes of 13 patients (42.11%). Patients with noninvasive FSS showed significantly greater improvement in visual acuity (17 eyes, 70.8%) than those with invasive FSS (5 eyes, 35.7%) (*P* = 0.026). Univariate and multivariate GEE model results revealed that sphenoid sinus wall sclerosis on CT was an independent factor for better visual prognosis (odds ratio = 4.434, 95% confidence interval (CI) 1.032–19.059, *P* = 0.045) (Table 3).

**Table 3:** Visual prognosis of fungal sphenoid sinusitis.

Variable	Univariable		Multivariable	
	OR (95% CI)	*P* value	OR (95% CI)	*P* value
Gender (M:F)	0.481 (0.117–1.982)	0.481	–	–
DM	0.657 (0.169–2.549)	0.543	–	–
Initial BCVA ≤ 20/400	1.667 (0.449–6.193)	0.446	–	–
Disease duration ≤ 1 month	1.123 (0.305–4.135)	0.861	–	–
Microcalcifications	0.625 (0.157–2.485)	0.504	–	–
Sinus wall erosion	0.333 (0.082–1.361)	0.126	–	–
Sinus wall sclerosis	4.444 (1.118–17.668)	0.034*	4.434 (1.032–19.059)	0.045*
Pathologic diagnosis^a^	0.229 (0.056–0.931)	0.039*	0.229 (0.053–1.001)	**0.050**
Types of fungi	7.000 (0.700–70.045)	0.098	–	–

Significant values are in bold.

*The labeled variables were entered into multivariate regression analysis.

^a^The patients were diagnosed with invasive or non-invasive fungal sinusitis based on histopathological findings.

## Discussion

Fungal sinusitis is a relatively uncommon disease and is broadly classified into two categories: invasive and noninvasive^6^. In the present study, we found that FSS with ocular complications was more common among the older women with a mean age of > 62 years, similar to that described in previous reports^12^. This suggests that advanced age may be a risk factor for FSS.

Abnormal immune function is an important inducer of fungal invasion of the paranasal sinuses^13,14^. The typical patients with noninvasive FSS are immunocompetent, while affected patients with invasive FSS tend to be critically ill, and demonstrate some degree of compromised immune function, such patients typically include those with decreased host cell-mediated immunity, specifically with impaired neutrophil function, hematologic malignancies, aplastic anemia, hemochromatosis, poorly controlled diabetes, acquired immunodeficiency syndrome, or organ transplantation; or are undergoing immunosuppressive treatments, such as systemic steroids or chemotherapeutic agents. Infrequently, invasive FSS has been reported in patients with normal immune function^9^. In this study, 18 patients (56.25%) had a diagnosis of diabetes mellitus, 4 of whom had uncontrolled diabetes. In addition, one patient was on antibiotic therapy for chronic bronchiectasis and one was taking long-term corticosteroids for rheumatoid arthritis. We have assessed the neutrophil count of all patients to categorize the degrees of impaired immune function. However, the neutrophil count of all patients is within the normal range. Patients with uncontrolled diabetes are known to have poor humoral immunity and neutrophilic dysfunction, which provide a favorable environment for the fungus to thrive^15^. The long-term systemic use of antibiotics or corticosteroids is also an important risk factor for altered immune function. Although the neutrophil counts of these patients were within normal limits, we suspected that impaired neutrophil function could be the main reason for the poor immunity, which reminded us to pay attention to neutrophil function in our future work.

In our study, 13 eyes had NLP, of which nine exhibited fungal invasion of the mucosa or submucosal tissue of the sphenoid sinus on histopathology. The other four fungal ball or allergic fungal rhinosinusitis were accompanied by infiltration of acute and chronic inflammatory cells. These findings suggest that the mechanism of visual impairment in FSS sinusitis may be related to the direct invasion of fungi or the local immune response caused by the fungal infection.

It is generally not difficult to locate the lesion at the orbital apex or cavernous sinus^16^ when vision loss coincides with binocular diplopia, motility deficits, ptosis, or exophthalmos, the diagnostic process becomes more straightforward, typically involving the exclusion of tumors. Sinonasal malignancy is always with a short history and rapid progression, commonly arise from the maxillary sinus. CT scan shows a soft tissue mass filling the sinus cavity and extensive osteolytic destruction of the sinus wall. The masses have uneven density and irregular morphology^17^. However, if there is only headache or ocular pain followed by monocular or binocular vision loss, it is easy to misdiagnose as noninfectious optic neuritis, which is a relatively common disease, and administer high-dose glucocorticoids as treatment. In our study, four patients were treated with glucocorticoids preoperatively. Visual acuity of three patients did not show any improvement, while the other one's visual acuity further decreased after glucocorticoids treatment. For these patients, there is a potential risk of infection spread and, even fatal consequences. Fortunately, there were not any other complications due to the glucocorticoid therapy in these four patients, and they were correctly diagnosed in time during the subsequent treatment. Therefore, in order to avoid the serious consequences caused by misdiagnosis, for older women with decreased vision and persistent headache, especially those with diabetes, orbital or cranial neuroimaging examinations should be performed to exclude the FSS.

CT typically is effective in delineating the fine bone structures and intra-sinus calcification^18,19^. Noninvasive FSS is mainly characterized by calcification, bone thickening, and sclerosis of the sinus wall. Conversely, invasive FSS usually causes bone destruction of the sinus, but calcification is rare. MRI is useful for diagnosing and identifying FSS, as it provides better details of lesions of the orbital apex, cavernous sinus, brain parenchyma, and pterygomaxillary fossa. In this study, noninvasive FSS showed marked hypointensity on T2WI, which may be related to heavy metals, such as calcium salt, iron, and magnesium in fungal secretions^20^. The manifestations of invasive FSS are different from those of noninvasive FSS on T2WI. The invasive FSS showed variable signals on T2WI, which was related to the course of the disease. The early stage is dominated by hyperintensity and the late stage by hypointensity, but it is usually uneven. Histopathology showed tissue necrosis with varying numbers of inflammatory cells in the early stage, some cases formed granulomas, and there was prominent fibrosis in the late stage. Most of them were accompanied by peripheral mucosal inflammation, manifested as mucosal thickening, and appeared isointense on T1WI and hyperintense on T2WI, the edge was evidently enhanced, which is valuable to distinguish from noninvasive FSS.

Surgical exenteration and reconstruction of a sufficient sinus cavity are effective treatments^21^. All patients in this study underwent surgical debridement, and 13 were treated with antifungal drugs after exenteration. All patients survived, except three patients with Mucor infection died of subsequently intracranial dilation at 1, 3, and 5 months postoperatively, respectively. Cavernous carotid artery occlusion was identified in two patients and Mucor meningitis was identified in the other one. These three patients had a diagnosis of diabetes mellitus. Aspergillus and Mucor are saprophytic fungi, which thrive in an environment of low oxygen tension and hyperglycemia^21^. Both have an affinity for invading blood vessels, however, Mucor has been suggested to be more aggressive with a strong affinity for arteries^15^. It can grow along the vascular wall or into the lumen, leading to fungal vasculitis and thrombosis^22^.

Visual acuity was improved after treatment. The overall improvement rate of visual acuity was higher in noninvasive FSS than in invasive FSS. However, there was no correlation between fungal invasion and visual prognosis in multivariate analysis. We noted that the *P* value was exactly 0.05 in multivariate analysis, which may have resulted from the sample size limitation, and future studies with larger sample sizes may have led to different conclusions. In addition, sphenoid sinus wall sclerosis on CT was an important independent factor affecting visual prognosis. Remodeling of the sphenoid sinus wall is due to stimulation of fungal reproduction or secondary chronic inflammation, indicating that the pathogen has low virulence and poor invasiveness and causes less damage to the optic nerve. Therefore, the vision of some patients will be improved to a certain extent.

This study had some limitations. First, this was a single–center retrospective study. Second, our study lacked other visual function evaluation methods, such as the visual field. Therefore, these assessment methods should be included in future prospective studies.

## Conclusion

Fungal sinusitis, although relatively rare, can cause varying degrees of visual impairment. When symptoms are nonspecific, it can be misdiagnosed as noninfectious optic neuritis and inappropriate treatment with high-dose glucocorticoids may lead to infection spread, even fatal consequences. Immediate imaging of the orbit and sinuses is imperative to avoiding misdiagnosis and prompt initiation of appropriate treatment as soon as possible is essential for the preservation of visual function. In severe cases, especially in invasive FSS, early endoscopic sphenoidotomy and antifungal therapy are effective treatments.

## Data Availability

The datasets used and analyzed during the current study available from the corresponding author on reasonable request.

## Abbreviations

FSS: Fungal sphenoid sinusitis
AFS: Allergic fungal sinusitis
DR: Diabetic retinopathy
BCVA: Best-corrected visual acuity
log MAR: Log of the minimum angle of resolution
CF: Count fingers
LP: Light perception
NLP: No light perception
CT: Computed tomography
MRI: Magnetic resonance imaging
T1WI: T1-weighted images
T2WI: T2-weighted images
GEE: Generalised estimating equation
CI: Confidence interval
OR: Odds ratio
